# Process optimization with acid functionalised activated carbon derived from corncob for production of 4-hydroxymethyl-2,2-dimethyl-1,3-dioxolane and 5-hydroxy-2,2-dimethyl-1,3-dioxane

**DOI:** 10.1038/s41598-021-87622-z

**Published:** 2021-04-21

**Authors:** Jaspreet Kaur, Anil Kumar Sarma, Poonam Gera, Mithilesh Kumar Jha

**Affiliations:** 1 Department of Chemical Engineering, Dr. B. R. Ambedkar National Institute of Technology, Jalandhar, Punjab India; 2 Chemical Conversion Division, Sardar Swaran Singh National Institute of Bio-Energy (An Autonomous Institute of MNRE, Government of India), Kapurthala, Punjab India

**Keywords:** Renewable energy, Environmental sciences, Materials for energy and catalysis

## Abstract

In this article, a two-step activated carbon preparation technique from corncob has been elucidated. The derived catalysts AAC-CC has been characterized using various techniques for the determination of their structural properties and compared with AC-CC, already reported with another article. The conjugated boat structure of AAC-CC resulted in a very high surface area (779.8 m^2^/g) and high pore volume (0.428 cc/g). This unveils the suitability of AAC-CC as better among the two catalytic pathways for solketal production. The activated carbons so prepared have been used for the valorization of glycerol to produce 2,2-Dimethyl-1,3-dioxolane-4-methanol (solketal), oxygenated additives to fuel. The face-centered composite design (FCCD) of RSM was applied for the optimization of the reaction parameters for the ketalisation reaction using AAC-CC as a catalyst. From the optimized results, the acidic catalyst AAC-CC resulted in a glycerol conversion, i.e. 80.3% under the actual laboratory experiment. Moreover, the catalyst could be reused for three consecutive batch reactions without (< 5%) much reduction of activity and no distinctive structural deformity.

## Introduction

Biofuels are the leading contenders and are fossil fuel alternatives that are made from organic material. Biofuels can work as an alternative replacement to energy needs from vehicle fuel to central home heating^1^. Though we can use conventional fuels in the transport sector in diesel engines, the use of biodiesel as a renewable fuel which is an alternative to petrodiesel has been recognized as an important transition in the liquid fuel-based locomotives, economy, and environment^2,3^. Various physicochemical properties that differentiate both conventional fuels and biofuels are shown in Table 1. Density, kinematic viscosity, heating value, acid value, cloud point, flash point, etc. are a few such important properties^3^.

**Table 1:** Comparison of physicochemical properties, merits and demerits of biofuels and conventional fuel^3,4^.

Fuel	Density [kg/dm^3^] at 15 °C	Kinematic viscosity [mm^2^/s] at 40 °C	Flash point (closed cup method) [°C]	Oxygen content [w%]	Merits	Demerits
Diesel	0.837 (kg/dm^3^) at 15 °C	2.98	70	0	More efficient, longer life span, lower CO_2_ emission	High sulphur content
Biodiesel	0.905 (kg/dm^3^) at 15 °C	6.43	221	13.92	Higher flash point, higher cetane number, ultralow sulfur content, better lubricity, improved biodegradability, and a smaller carbon footprint	Couldn’t be used at lower temperatures, expensive than diesel
Vegetable Oil	855–930 (kg/m^3^) at 40 °C	3.7–5.8	120–243	-	Low sulphur and no aromatic content, biodegradability, renewability	Low calorific value

The main source of glycerol production is the transesterification process of lipid (oil/fat) during biodiesel production^5^. One mole of glycerol is formed as a by-product from one mole of triglyceride (a lipid molecule) in addition to the three moles of biodiesel. The crude glycerol obtained from biodiesel plants comprises a large number of impurities and other chemicals, for instance, methanol, organic and inorganic salts, water, vegetable colours, mono, and di-glyceride traces and soap^6,7^. The superabundance of glycerol from biodiesel manufacturers will lead to the availability of glycerol at a demean price; therefore, it would create a huge market value for the applications of crude glycerol^8^.

**Figure 1:**
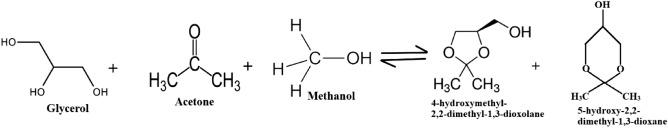
Ketalization reaction for the production of ketals from glycerol.

Thus, upgradation must be needed in the processes for transforming glycerol into a pure form or to other value-added products. The glycerol obtained from the biodiesel industry can be a game-changer if the same is properly processed for value-added chemicals/derivatives. A lot of commodity chemicals can be derived from a highly functionalized glycerol molecule such as di-tertiary butyl (DTBG) and tri-tertiary butyl ether (TTBG) from etherification, glycerol mono-, di-, and tri-oleate (GMO, GDO, and GTO) by esterification, glycerol carbonate by carboxylation, acrolein from dehydration, propanediol from hydrogenolysis and hydrogen via glycerol reforming using various catalysts derived from waste biomass materials^9,10^. One plausible pathway that the glycerol can be converted to five and six-membered oxygen-containing functionalized cyclic compounds reported by several authors is the production of 2,2-Dimethyl-1,3-dioxolane-4-methanol (solketal) via ketalisation. The chemical reaction for the production of two ketal species is shown in Fig. 1^11^. Solketal is a ring-shaped di-ether that has an additional hydroxyl group^12^. It can be used as an additive in gasoline, diesel, or biodiesel to increase ignitability and reduce particulate emission^12^.

There are various commercially developed catalysts reported in the literature having different physicochemical properties responsible for the conversion of glycerol to solketal. The nature of these catalysts may be either acidic or alkaline and the reactions may be either homogeneously or heterogeneously catalyzed^13^. Oprescu et al. reported the green solid superacid catalyst SO_4_^2−^/SnO_2_ for the ketalisaton of glycerol to produce solketal with a yield of 97.5%^14^. Rodrigues et al. reported the activated carbons produced from olive stone functionalized with acid groups for solketal production with 97% conversion of glycerol^15^. The Nb–SBA-15 (Nb metal modified SBA-15 catalyst) gives 95% glycerol conversion as reported by Ammaji et al.^16^. Fe(NO_3_)_3_·9H_2_O exhibited the highest catalytic activity, virtually converting all the glycerol to solketal, with 95% selectivity as reported by Silva et al.^17^. The OTS-grafted HY (organosilane modified HY) catalyst reported having high catalytic activity (89% conversion) at low temperatures as stated by Rahaman et al.^18^. Shirani et al. reported a heterogeneous resin catalyst, i.e., Purolite PD206 for glycerol conversion to solketal with a 95% yield^19^. A mesoporous phenol sulfonic acid-formaldehyde polymeric acid catalyst was synthesized that provides glycerol conversion of 97% as reported by Laskar et al^20^. BEA zeolite and the hierarchical zeolite of MFI structure exhibited high catalytic activity in solketal production, i.e. almost 85% of glycerol conversion and 98% selectivity to solketal were achieved as reported by Kowalska-Kuś et al^21^.

Various acidic or basic homogeneously or heterogeneously catalyzed conversion processes amongst the reported literature unveil that basic catalysts are considered less active as they provide a lower conversion as compared to the acidic catalysts. As per the literature, the basic oxide catalysts partially leached during the reaction which hinders the separation of the catalyst from the reaction mixture^22^. Also, base catalysts can’t give high conversion at lower temperature and for a shorter reaction time that led to the formation of salts^23^. Thus, the acidic catalysts show the highest catalytic activity as compared to the basic one and lead to better conversion due to the availability of the more active sites to the catalyst that will help in the reaction for higher conversion.

Physical activation, chemical activation, physical–chemical and microwave-assisted activation are the common activation methods for the preparation of catalysts^24^. Physical activation consists of heat and steam, chemical activation involves activation using chemical agents, physical–chemical activation involves both physical and chemical activation and microwave-assisted activation is done by microwave radiation^25^.

It has been observed from the various reported literature that very few renewable precursors-based catalysts are developed^26^. Among all the catalysts investigated, acid and base activated carbons derived from agricultural waste, i.e. corncob has not been reported yet for the production of solketal. Keeping in view the introduction of cleaner production technology for solketal production using renewable precursor-based catalysts, a locally available, merely a waste lignocellulosic residue corncob was studied for the production of acid and base activated carbon catalyst and reported in this work. This lignocellulosic material consists of cellulose of 40–44%, hemicellulose of 31–33% and lignin of 16–18^27^.

Although it is challenging to use biomass in energy applications due to the NO_x_ emissions during thermal processing of biomass via pyrolysis, there are several ways available for their migration. Some of the possible ways for their migration are chemical reduction of NO_x_, NO_x_ oxidation, using a sorbent in the combustion chamber etc.^28^. Thus, using waste biomass as renewable precursors proves to be a cost-effective method.

Complete physicochemical characterization of the prepared acid-activated carbon (H_2_SO_4_) (AAC-CC) has been accomplished, presented and discussed in this article. Process optimization under different sets of reaction conditions using response surface methodology (RSM) for ketalisation reaction using AAC-CC has also not been reported elsewhere except this article along with optimal production of 4-hydroxymethyl-2,2-dimethyl-1,3-dioxolane and 5-hydroxy-2,2-dimethyl-1,3-dioxane using virtual laboratory setup.

## Results

### Characterization of catalyst using various physical and adsorption techniques

The plausible structure of the derived catalyst is shown in supplementary section II (SS-II).

The surface groups present in the carbon material produced were characterized and identified using different techniques before their utilization for the ketalization reaction. The presence of different functional groups in the structure was identified from an extensive study of FTIR and XPS analysis. The XRD patterns of the catalyst showed the amorphous nature of the carbon material.

**Figure 2:**
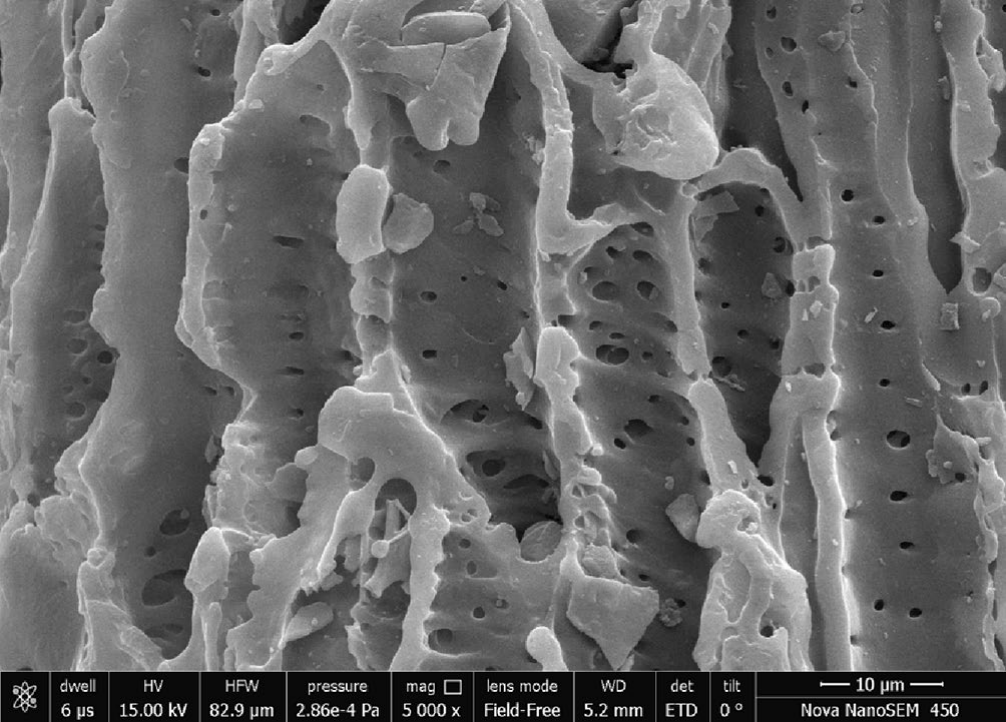
SEM images of derived catalysts Acid Functionalized Activated Corncob.

The microstructural evaluation was done using Scanning Electron Microscopy (SEM) by doing the sputter coating of the sample before the SEM analysis to make the sample conducting. This step involves the coating with a conductive material like gold for obtaining better quality images. SEM micrographs of the derived functionalised activated carbon have been shown in Fig. 2. It can be observed from the picture that AAC-CC has developed a conjugated boat-like structure having a rough or uneven surface with cavities and cracks in the external surface and random pore size distribution^29^. The elemental analysis was performed using energy-dispersive (SEM–EDX) and the elemental composition of the sample showed the presence of Carbon, Oxygen, Sulphur in AAC-CC. Carbon is present in the highest amount i.e. 90.99% in AAC-CC responsible for the higher glycerol conversion^24^. Thus, carbonization is confirmed in sample^30^ (Table 2) with minimum carbon loss in AAC-CC. The sputter coating does not affect the elemental composition of the sample as the acid-activated carbon does not contain gold in it.

**Table 2:** Elemental analysis of the catalyst.

AAC-CC		
C	O	S
90.99	8.40	0.52

**Figure 3:**
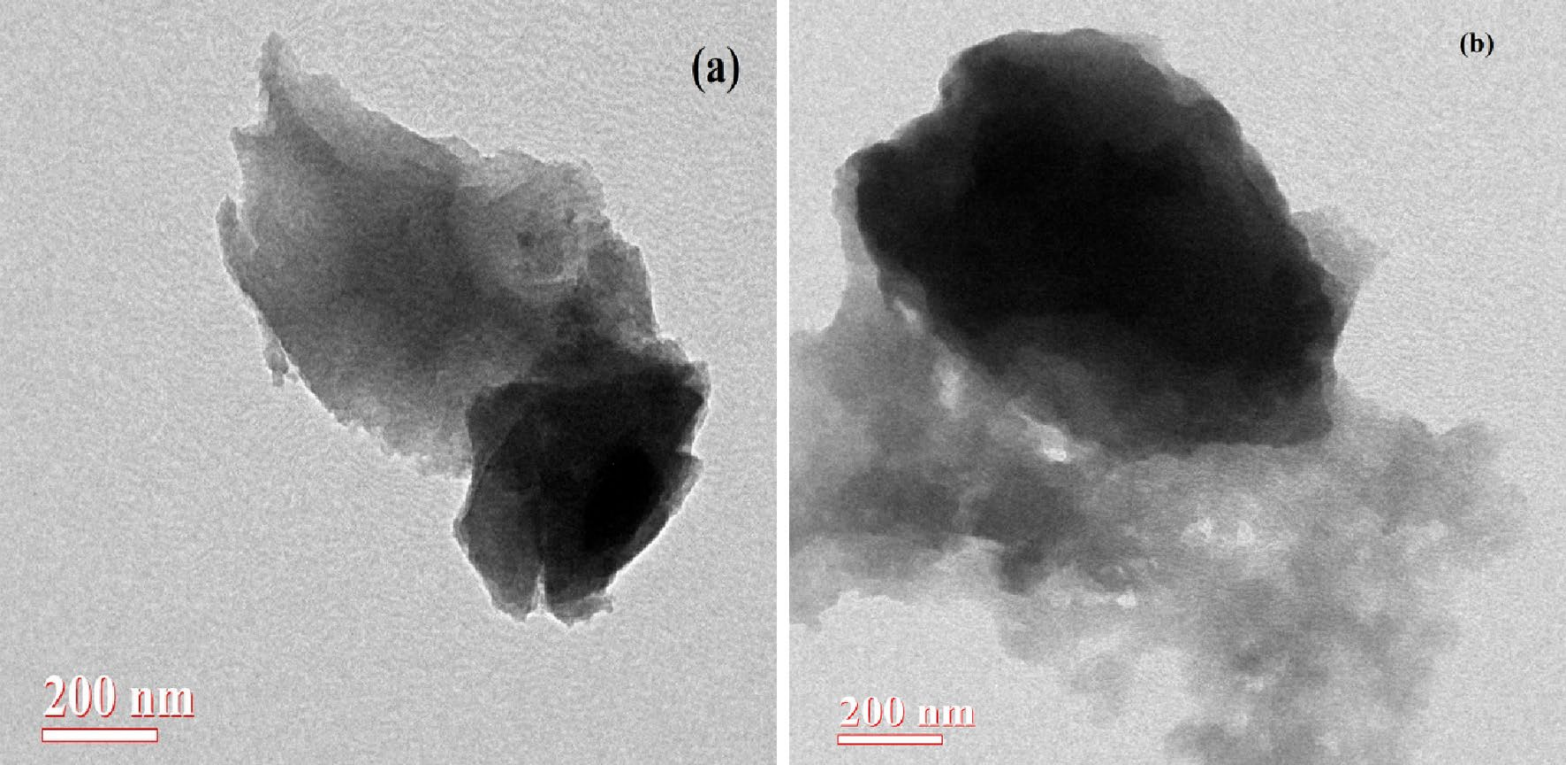
TEM images of the catalysts (**a**) AAC-CC (**b**) AC-CC^34^.

The TEM micrographs of the AAC-CC are shown in Fig. 3a. The TEM results are in good agreement with SEM results showing the porosity of the produced catalysts. The images revealed a complete porous carbon structure for both the activated carbons even at higher magnification. The existence of the pore structure of the catalyst is attributed to the NaOH activation adopted during the synthesis of the AC-CC catalyst^31,32^ (Fig. 3b) and H_2_SO_4_ activation AAC-CC as shown in the images^33^. The sizes and uniformity envisage that the glycerol molecule with a size of 0.5–0.6 nm can easily fit and escape from these pores where the active functional groups are present that facilitates the group transfer and cyclization reactions of glycerol.

**Figure 4:**
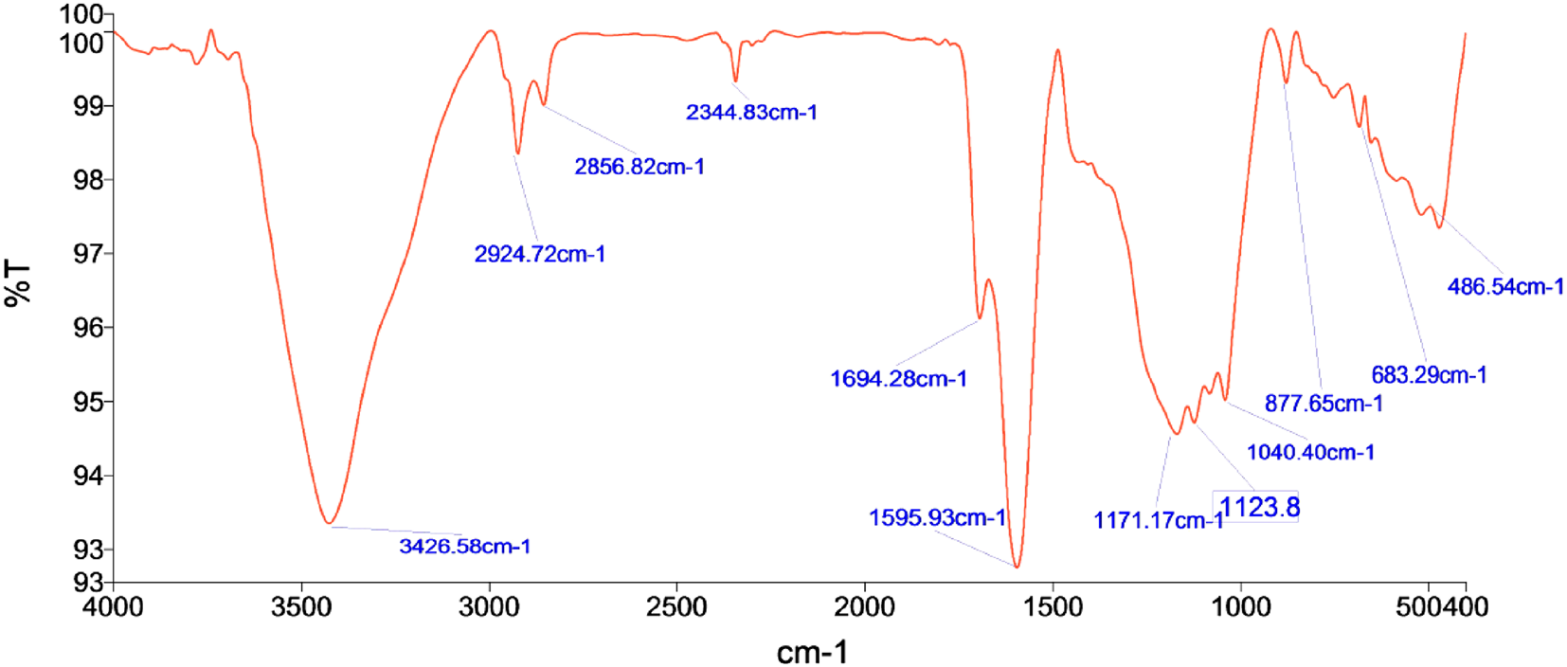
FTIR patterns of the derived activated carbons AAC-CC.

FT-IR spectra show a broad peak at around 3426.50 cm^−1^ in AAC-CC (Fig. 4) for stretching of –OH bond of alcohol. The 2924.72 cm^−1^ peak shows the aliphatic C–H stretching vibration in AAC-CC. The peak at 2856.82 cm^−1^ shows the vibration of the -OH group attached to ring carbon in the derived catalyst. There is a broad peak at 1694.28 cm^−1^ which shows the stretching of the C=C due to the influenced functionalities. The sharp band at 1595.93 cm^−1^ corresponds to a stretch probably from an amide in the acid-activated catalyst^35^. The peaks at 1457.57 cm^−1^, 1381.38 cm^−1^, and 1084.04 cm^−1^ show the C–C stretching vibration of the chain hydrocarbon parts due to different structural influences, C–H stretching vibration in alkanes or an alkyl group and C–S group, respectively in base-activated catalyst^34^. The peak at 779.62 cm^−1^ shows the absorption of the SiO_2_ and the 548 cm^−1^ peak shows the C–H stretching of aromatic compounds^34,36^. The additional bands at 1171.17 cm^−1^ and 1123.8 cm^−1^ show the stretching of sulfonated groups in acid activated carbon^26^. The functional groups represent the chemically active components of the catalyst that accelerates the rate of reaction.

The values of specific surface area calculated using the BET equation are presented in Table 3 together with the values of the total pore volume, Vp, taken at P/Po = 0.99, and average pore radius pore size distribution curves for activated carbon samples have been obtained from BJH calculation method^37^. The significance of the bold in Table 3 is that it refers the results of the present study while all other results are reported from literature.

**Table 3:** Comparison analysis of Surface area, pore volume of different activated carbons.

Derived catalysts-Activated carbon	Biomass	Specific surface area -A_BET_ (m^2^/g)	Pore volume—Vp (cc/g)	Activity (Conversion)	References
**Acid-Activated Carbon**	**Corncob—Agricultural waste**	**779.831**	**0.428**	**80.3%**	**This study**
Base-Activated Carbon	Corncob—Agricultural waste	13.901	0.011	72.12%	^34^
Activated Carbon	Potato peel waste	833	0.44	84%	^39^
Activated Carbon	Poultry litter	1129.5	–	–	^40^
Activated Carbon	Straw pellets	1349.6	0.69	66–100%	^41^
Activated Carbon	Wood strips	1194.4	0.61	66–100%	^41^
Activated Carbon	Date seeds	422.9	0.16	–	^42^
Activated Carbon	Pine wood	969.075	0.5	–	^43^

**Figure 5:**
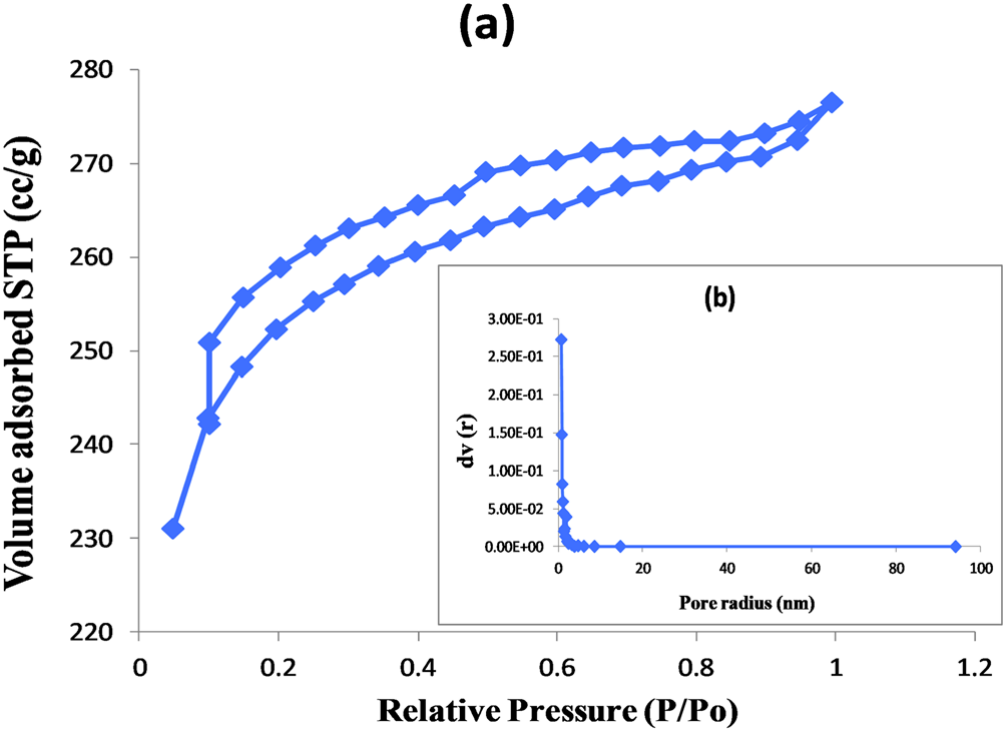
For acid activated carbon (**a**) N_2_ adsorption–desorption isotherm (**b**) Pore size distribution.

The adsorption–desorption isotherm of AAC-CC is classified as type IV and reveals a hysteresis loop of H4 type implies to narrow slit pores including the pores in the micropore region as shown in Fig. 5^38^. H4 loops are often found with micro-mesoporous carbons.

A very high surface area and higher pore volume of AAC-CC unveil its suitability as the better of the two catalytic pathways during the solketal production as shown in Table 3.

**Figure 6:**
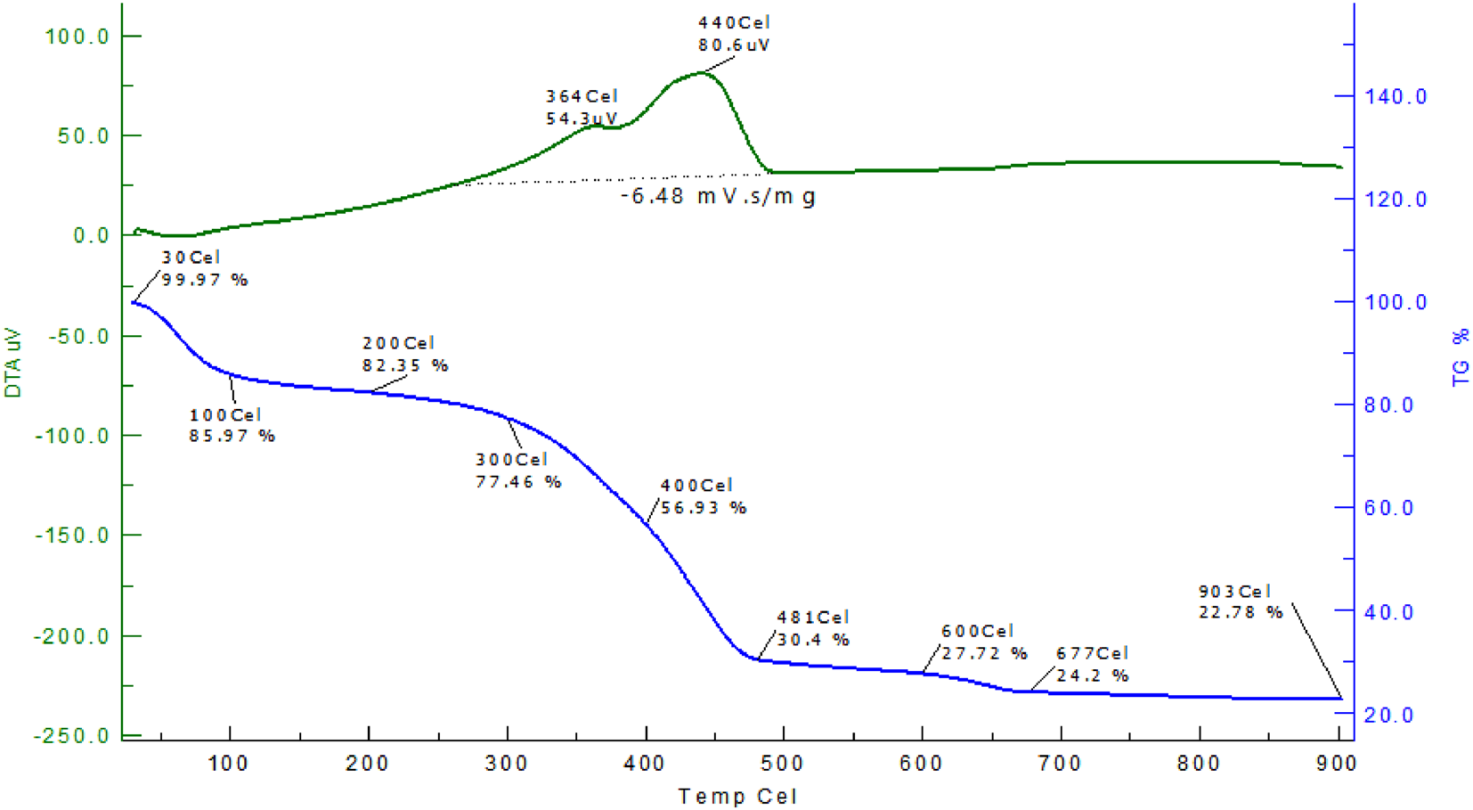
TG–DTA curve of the AAC-CC catalysts.

The thermal behaviour of the AAC-CC (TG and DTA) was observed in the temperature range from 30 to 900 °C. TG and DTA curves show the weight loss from 30–903 °C for AAC-CC as shown in Fig. 6. The weight loss at the initial stage up to 100 °C shows the removal of moisture while volatilization occurs at about 150 °C in AC-CC^34^. In AAC-CC, the weight loss from 200–300 °C corresponds to the decomposition of the sulfonated groups. The weight loss from 300–400 °C is due to the loss of cellulosic materials. The prominent desorption occurs between 400–481 °C^44^. The weight loss at 600–700 °C shows the decomposition of the produced carbon and the weight loss at 800–900 °C shows the decomposition of the organic components. After 600 °C, the fusion of the carbon occurs till 800 °C and 800–900 °C is the ash fusion temperature.

**Figure 7:**
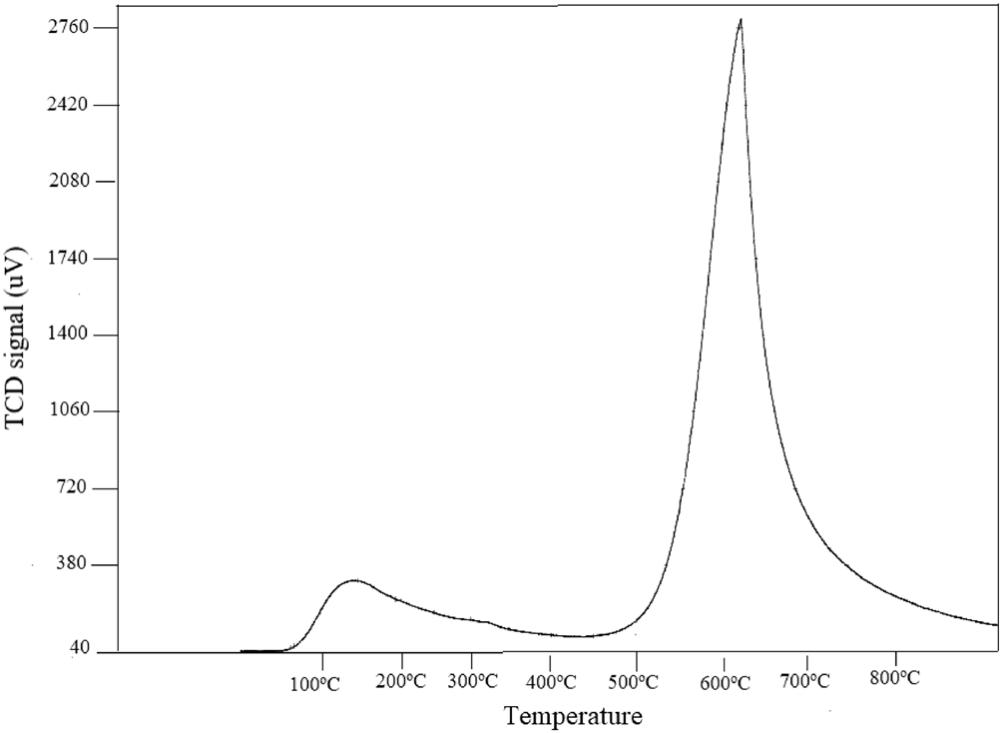
NH_3_-TPD of acid activated carbon AAC-CC.

Although Boehm titration (BT) is also a method to determine quantitatively the oxygen-containing groups in activated carbons, simple and cost-effective, it is time consuming and variations while opting measuring protocol that influences the results of analysis^45^. Though TPD and BT provide similar trends in results, a number of groups determined by TPD is higher than those obtained by BT. BT cannot determine the groups that can form on the surface such as ketones, ethers, aldehydes, and pyrones, groups that contain nitrogen, phosphorus, or sulfur^46^. Thus, the acidic sites of the catalysts are determined by NH_3_-TPD as these are the active sites responsible for the conversion of glycerol to solketal at a modest temperature^47^. The acidic strength of solid acid catalysts in the NH_3_-TPD profiles can be classified into three regions depending on their strength in the temperature range. These acidic sites are denoted as weak (150–300 °C), moderate (300–450 °C), and strong (450–650 °C)^16^. The desorption peak of NH_3_-TPD for AAC-CC shows a weak acidic site at 147.1 °C, while showing a sharp peak at 605 °C with higher intensity having strong acidic strength (Fig. 7). The total acidic amount of 0.187 mmol/g was obtained for the acidic site.

**Figure 8:**
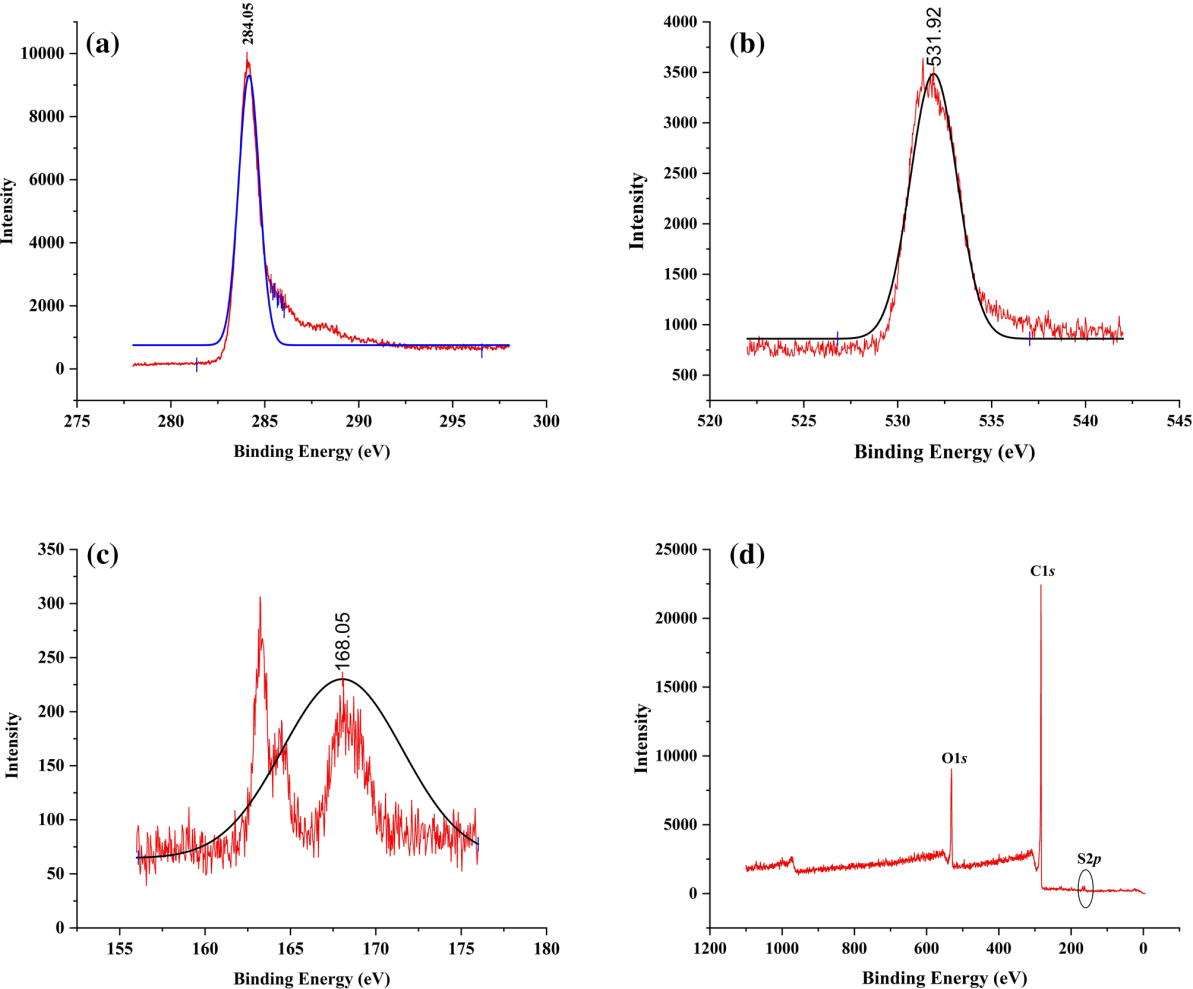
Deconvolution of (**a**) Carbon, (**b**) Oxygen, (**c**) Sulphur and (**d**) XPS spectrum.

The characteristic peaks and their confirmation showing the presence of carbon, oxygen and sulphur groups attached with the AAC-CC and the XPS spectra showing the surface chemistry are shown in Fig. 8a,b,c,d. XPS detects the composition of the species on the surface and their binding energies, along with the oxidation states^39^. The main C*1s* peak of AAC-CC was observed at the binding energy of 284.5 eV belongs to C-O and one at 288.4 eV belongs to C–C bonding. The signals at 531.95 eV of O*1s* are associated with oxygen-carbon bonds (C=O). The XPS analysis confirms the presence of –SO_3_H groups, based on a Sulphur peak at ~168.11 eV. The results of characterisation and the elemental analysis obtained by XPS of the groups present in AAC-CC are listed in Table 4.

**Table 4:** XPS data for AAC-CC.

Relative amount (%)			Atomic Ratio	Deconvolution of C_1s_, O_1s_ and S_2p_		
C_1s_	O_1s_	S_2p_	O/C	C_1s_	O_1s_	S_2p_
88.19	9.60	0.14	0.002	284.05 eV	531.95 eV	168.11 eV

Finally, a comparison of the physicochemical characteristics of both the catalysts viz. AC-CC and AAC-CC has been put together in Table 5. It is clearly established from the table that AAC-CC has better catalytic properties than AC-CC.

**Table 5:** Comparison of properties of base-activated and acid-functionalised activated carbon.

Name of catalyst	Properties				
	Structure	Elements present	FTIR peaks	Pore radius (nm)	Acidic strength
AC-CC (base-activated)	Rib shaped	Carbon, Oxygen, Sodium, Silicon, Sulphur	At 1607.50 cm^−1^ for carbon bonds, 779.62 cm^−1^ for SiO_2_ absorption	1.223	Weak
AAC-CC (acid-functionalised)	Conjugate boat shaped	Carbon, Oxygen, Sulphur	1694.28 cm^−1^ for carbon stretching, 1171.17 cm^−1^ and 1123.8 cm^−1^ for sulfonated groups	0.001	Strong

## Discussions

### Catalyst Activation and functionalization

**Figure 9:**
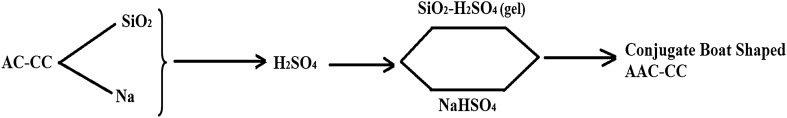
Basic reaction of AC-CC to produce AAC-CC.

The carbon percentage in AC-CC was 78%, whereas in AAC-CC it is 91%. Moreover, the sodium, oxygen and silica content are drastically reduced in the AAC-CC. This is confirmed from the EDX and XPS data that the percentage of C increases by 13% after acid functionalization of the NaOH activated carbon. Moreover, the Rib shaped AC-CC has been converted to a conjugate boat structure in AAC-CC, resulting in high porosity and 50–60-fold increased surface area. This may be attributed to the basic reactions for the removal of silica and Na bonded with AC-CC in step -II production of activated carbon as shown in Fig. 9.

### Effect of reaction parameters on solketal production

In the present work, Response Surface Methodology (RSM) was employed to optimize the values of process parameters for the ketalization reaction to get the improved glycerol conversion with derived catalysts by correlating the experimental and predicted values; numerical optimization method and analysis of standard error in the obtained values; quadratic regression in the model through desirability function as 0.999 for the precision of the results. Also, possible interactions between process factors were determined for their overall influence on the progress of the process. After completion of optimization of the process via RSM, the obtained 30 sets of experiments were conducted in actual laboratory conditions for the production of solketal. The obtained results are summarised in Table 2 in the results and discussions in the supplementary information (S-II) in the form of observed values of RSY against the coded values.

The effect of reaction parameters (molar ratio, time, temperature and catalyst amount) on RSY were also studied by plotting three-dimensional (3-D) surface curves shown in S-II, Fig. 6. The conversion of glycerol via ketalisation reaction was observed to be influenced by parameters within the range: molar ratio (1:2:2 to 1:8:8), time (1 to 3 h), temperature (50 to 150 °C) and catalyst amount (1 to 5 wt.%). The highest value of RSY was found to be 80.3% against the predicted value of 73.807% for solketal production corresponding to the parameters with molar ratio-1:8:8, for 2 h at 100 °C and 3wt.% catalyst loading. It was observed that a further increase in the reaction temperature associated with lower glycerol conversion because high temperature is associated with a higher rate of dissociation and lower stability of the product and was confirmed in Table 2 in S-II. Also, solketal production is an exothermic reaction and an increase in temperature reduces the product formation. From the shape of the 3-D surface plots, it was concluded that the effect of molar ratio on glycerol conversion dominates when compared with the effect of other variables like conversion increases with an increase in molar ratio but time has not shown a significant change in conversion as shown in Fig. 6a, S-II. These results have been found in concordance with the optimized results obtained by RSM employing Analysis of Variance (ANOVA).

The catalytic activity was also studied for the ketalization reaction i.e. conversion of glycerol to solketal using the derived functionalised catalyst obtained from corn cob. To check the stability of the catalyst and effectiveness of the active sites of the catalyst, a reusability study was carried out^34^. The catalyst was separated from the reaction mixture after the completion of the reaction by filtering the reaction mixture using filter paper. It was then washed with ethanol 3–4 times and then dried in an oven at a temperature of 90 °C. The study showed that the catalyst can be reused up to three consecutive batch reactions without much loss of the activity (< 5%) and no distinctive structural deformity. This shows the utility of the derived catalyst.

## Conclusions

Conversion of glycerol to solketal using acid-functionalized activated corncob-based carbon as a catalyst is a greener pathway and possible. The acid-activated carbon has a higher surface area, pore-volume, and strong acidic sites that catalyze the reaction with a higher conversion rate as compared to alkali-activated carbon. The molar ratio of the reactants (glycerol: acetone: methanol) has a significant effect on the ketalization reaction. The derived activated carbon can be used for hydrogen storage and electrode fabrication material for the fuel cells. It can be reused to produce various valuable chemicals from glycerol and a prototype can be developed in near future for cleaner production of solketal from crude glycerol.

## Experimental section

The chemicals used were of analytical grade. Glycerol (Sigma Aldrich—97% purity), Methanol (Sigma Aldrich—99.5% purity), Acetone (Sigma Aldrich—99.5% purity) were procured from the Merck Specialists Limited, Mumbai in the chemical conversion division of Sardar Swaran Singh National Institute of Bio-energy, Kapurthala, India. The corn cob used for the preparation of the catalyst was collected from the local mandi of the Kapurthala, Punjab, India.

### Catalyst preparation

The activated carbon was prepared using two-step activation methods by following the protocol as described below:

Step I: The received corncob was dried in the open air for 2–3 days to remove the excess moisture. The dried corncob was milled into small pieces of 75 mesh sizes. The ground corncob was calcined in the muffle furnace using the prepared crucible to provide the inert atmosphere during calcination. The calcination temperature was kept at 500 °C for 1–2 h. The calcined biomass was activated using NaOH as an activating agent with a molar ratio of 1:3 (Biomass: NaOH). After preliminary investigation and experimentation with the different impregnation ratio, 1:3 molar ratio (Biomass:NaOH) was adapted in this process as a higher ratio of activating agent develops AC with higher pore volume until optimal range^48,49^. The activated carbon produced was again heated in a muffle furnace at a temperature of around 450–500 °C. After heating the activated carbon, a suspension was obtained which was then filtered using Whatman filter paper while washing it continuously with distilled water to remove the excess alkali present in it.

Step-II: The filtered activated carbon was then dispersed in a beaker of 1000 ml containing concentrated sulphuric acid (2 M) solution. The activated carbon remains dipped in the acidic solution for the proper functionalisation of the catalyst for 8–10 h with proper stirring of the solution at an RPM 200. It was then filtered using Whatman filter paper. The collected functionalised carbon was then heated in an oven at 80–100 °C for 8–10 h for removal of the moisture. It was then stored in an airtight bottle which was used as an acid-activated carbon catalyst (labelled as AAC-CC) for further characterization and ketalization reactions.

**Figure 10:**
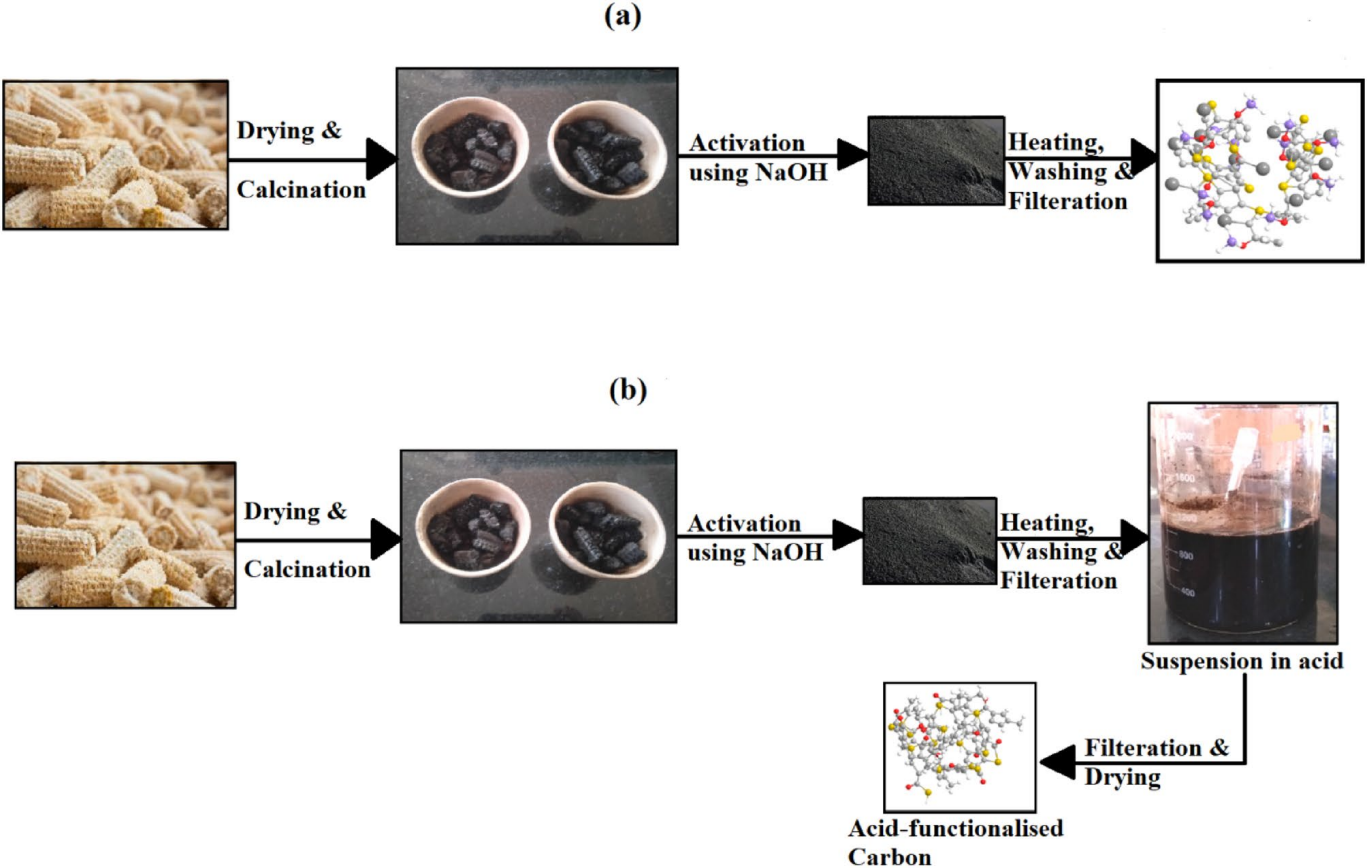
Synthesis scheme of produced (**a**) base-activated carbon, (**b**) acid-activated carbon.

The schematic representation of the synthesis of base-activated carbon and acid-activated carbon is shown in Fig. 10.

### Ketalization reaction

The reaction was carried out in a batch reactor as shown in the experimental section (S-II) in the supplementary information. It consists of three round neck flasks of 250 ml with varying reaction parameters such as the molar ratio (glycerol:acetone:methanol), reaction temperature, reaction time, catalyst loading (wt.% w.r.t. glycerol).

The flask was then kept in a beaker of 1000 ml containing water which acted as a water bath. This beaker was then placed on a magnetic plate with a stirrer and temperature controller to control the temperature and uniform mixing of the reaction mixture during the reaction. Two necks of the flask were blocked by a flask glass stopper and in one reflux and condenser were also attached to the flask with the help of the burette stand, to maintain the uniformity of the reaction mixture as methanol and acetone were volatile and may evaporate. The reaction was preceded at 600 revolutions per minute.

The reactions set were performed as per the process optimization described below.

### Process optimization

The ketalization reaction of the glycerol using AAC-CC was optimized using face centred composite design (FCCD) of RSM, Design-Expert software version 8.0 (STAT-EASE Inc., Minneapolis, USA) in terms of process parameters, the molar ratio (X1); time (X2); temperature (X3); catalyst amount (X4) to provide us with the maximum glycerol conversion (RSY) as a response (Y)^50^. Software provided 30 no. of reactions consisting of 24 non-central and 6 central axial points (α = 1). Central (axial) points were defined for the authenticity of the model via pure error variance. After completion of each reaction, the samples were collected in microcentrifuge tubes and then centrifuged using a 5430 R centrifuge to remove the traces of catalyst present in the sample. The centrifuged samples were then diluted 100 times and were analyzed using high-performance liquid chromatography (HPLC—Agilent Technologies model 1260 Infinity and glycerol conversion was calculated using equation (Eq. 1), from the graph obtained from HPLC.

1 $${\mathrm{Glycerol\,Conversion }}\left( \% \right) = \left[ {\frac{{{\mathrm{Peak\,of\,product}}}}{{{\mathrm{Peak\,of\,reactant}} + {\mathrm{Peak\,of\,product}}}}} \right] \times 100$$

### Catalyst characterization

The structural properties of the AAC-CC were characterized using various techniques such as Fourier Transform Infrared Spectroscopy (FTIR), Thermogravimetric Analysis (TGA), X-ray Diffraction (XRD), Surface area analysis (BET), X-ray Photoelectron Spectroscopy (XPS), Field Emission Scanning Electron Microscopy (FESEM), Transmission Electron Microscopy (HRTEM) and Temperature Programmed Desorption (TPD).

FTIR experiments were conducted to identify the functional groups. These were interpreted using a PE IR SUBTECH SPECTRUM ASCII PEDS 4.00 infrared spectrometer with a resolution of 1 cm^−1^. The materials were mixed with KBr powder pelletized and the pellets were scanned in the IR range from 4000 to 400 cm^−1^.

The carbonization behaviour of the AC-CC was determined by a simultaneous TG/DTA i.e. DTA with the proven capabilities of the TG measurement capabilities, providing thermal property information for a variety of samples. This was done using an SII 6300 EXSTAR thermal analyzer. Samples were heated in the temperature range from 35 to 900 °C at a constant heating rate of 5 °C/min in nitrogen with a 200 ml/min flow rate.

Nitrogen adsorption–desorption measurement was carried out using a BET surface area analyzer by St 2 on the NOVA touch 2LX instrument. The samples were degassed for 3 h at 180 °C on the degassed port. The linear part of the BET equation was used to determine the specific surface area.

The wide-angle X-ray diffraction pattern of the AC-CC was observed on Bruker XRD diffractometer using Cu-Kα radiation with a wavelength of 1.54 and F as a filter. The scanning angle (2θ) range was kept between 10° to 80°.

Scanning electron micrographs (SEM) and elemental analysis of the activated carbon were performed using Nova Nano FE-SEM 450 (FEI) coupled with an EDX analyzer of ultra-high-resolution characterization in an accelerating voltage of 15.0 kV.

Transmission Electron Microscopy (HRTEM) studies were done with a TEM TECNAI G2 20 S-TWIN (FEI) electron microscope. A drop of the powdered sample was dispersed into ethanol and then dropped onto a carbon-coated copper grid. The high resolution of the analyzer was provided with a voltage of 200 kV.

The presence of surface groups with Carbon, Sulphur, Oxygen, Silicon, and Sodium was identified and confirmed by X-ray photoelectron spectroscopy (XPS) using PHI 5000 Versa Probe III model.

The acidity of the catalyst surfaces was studied using Temperature program desorption (TPD) and performed using BEL’s new fully-automated catalyst analyzer (BELCAT II) with NH_3_ as investigating molecule and He as a carrier gas. The desorption of NH_3_ was performed after flushing using carrier gas up to temperature 600 °C at a heating rate of 10 °C/min.

## Supplementary Information

Supplementary Information
