# Supplementary material for: Process optimization with acid functionalised activated carbon derived from corncob for production of 4-hydroxymethyl-2,2-dimethyl-1,3-dioxolane and 5-hydroxy-2,2-dimethyl-1,3-dioxane
Source: Sci Rep. 2021 Apr 21;11:8567. doi: 10.1038/s41598-021-87622-z (PMC8060369; doi:10.1038/s41598-021-87622-z)
Supplement: Supplementary file 1 — Supplementary Information [file 41598_2021_87622_MOESM1_ESM.docx]

**Supplementary Information**

**Process Optimization with Acid Functionalised Activated Carbon derived from Corncob for production of *4-hydroxymethyl-2,2-dimethyl-1,3-dioxolane* and *5-hydroxy-2,2-dimethyl-1,3-dioxane***

Jaspreet Kaur^1^, Anil Kumar Sarma^2*^, Poonam Gera^1^, Mithilesh Kumar Jha^1^

^1^Department of Chemical Engineering, Dr. B. R. Ambedkar National Institute of Technology, Jalandhar, Punjab, India

^2^Chemical Conversion Division, Sardar Swaran Singh National Institute of Bio-Energy

(An Autonomous Institute of MNRE, Government of India), Kapurthala, Punjab, India

*Corresponding author: Phone: +919988425251, Email: [anil.sarma16@gov.in](mailto:anil.sarma16@gov.in)

**Experimental Section**

**Section I (S-I) – Catalyst preparation**

The filtered Ac is dispersed in a beaker of concentrated sulphuric acid (2M) solution is shown in figure 1(a). The prepared acid-activated carbon catalyst (labelled as AAC-CC) is shown in figure 1(b).

**
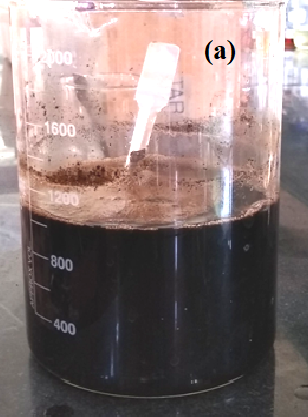

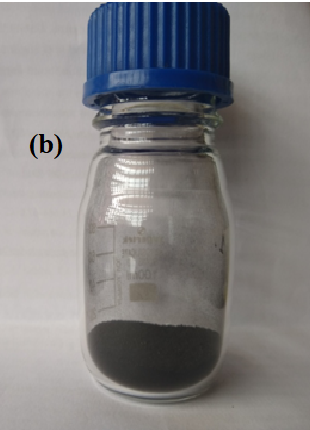
**

**Figure 1:** (a) Carbonised carbon after filtration dipped in the Sulphuric acid (b) Final acid functionalized activated carbon stored in the air tight bottle

**Section II (S-II) - Ketalisation reaction**

The batch reactor setup used for performing ketalisation reaction of glycerol with acetone and methanol is shown in figure 2.

**
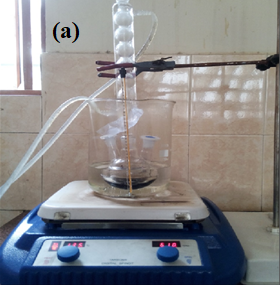

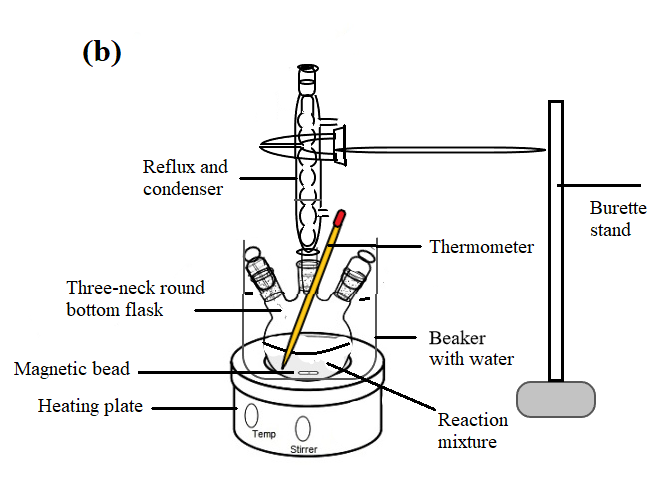
**

**Figure 2:** Batch reactor setup (a) and (b) Schematic representation

**Results and Discussions**

**Section I (S-I)**

The plausible structures of the single-molecule of the derived catalyst (Figure 3) and supramolecular structure (Figure 4) have been drawn using the ChemDraw software using the composition obtained from SEM-EDX analysis.

**
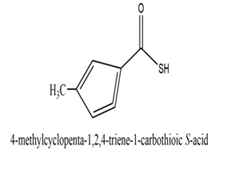
**

**Figure 3:** Plausible structure of a single molecule of Acid Functionalised Activated Corncob


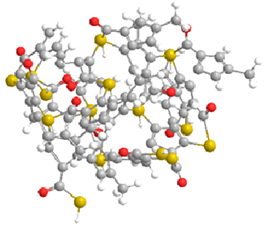


**Figure 4:** 3-D view of agglomerated structure of Acid Functionalised Activated Corncob

**Section II (S-II)**

The experimental data obtained from RSM was analyzed to obtain second order quadratic model equations of the dependent variable i.e. RSY in terms of coded and actual factors including their interactions using multiple regression analysis for fitness of the model (Equation 1).

**Eq 1:** $\mathbf{Y}\mathbf{=}\mathbf{16}\mathbf{.}\mathbf{64} \boldsymbol{\times}\mathbf{A} \mathbf{-}\mathbf{0}\mathbf{.}\mathbf{90611} \boldsymbol{\times}\mathbf{B} \mathbf{-}\mathbf{1}\mathbf{.}\mathbf{80278} \boldsymbol{\times}\mathbf{C}\mathbf{+}\mathbf{0}\mathbf{.}\mathbf{317778} \boldsymbol{\times}\mathbf{D}\mathbf{+}\mathbf{0}\mathbf{.}\mathbf{614375} \boldsymbol{\times}\mathbf{A} \boldsymbol{\times}\mathbf{B} \mathbf{-}\mathbf{0}\mathbf{.}\mathbf{56813} \boldsymbol{\times}\mathbf{A} \boldsymbol{\times}\mathbf{C} \mathbf{-}\mathbf{0}\mathbf{.}\mathbf{169438} \boldsymbol{\times}\mathbf{A} \boldsymbol{\times}\mathbf{D} \mathbf{-}\mathbf{1}\mathbf{.}\mathbf{07813} \boldsymbol{\times}\mathbf{B} \boldsymbol{\times}\mathbf{C} \mathbf{-}\mathbf{0}\mathbf{.}\mathbf{33438} \boldsymbol{\times}\mathbf{B} \boldsymbol{\times}\mathbf{D} \mathbf{-}\mathbf{0}\mathbf{.}\mathbf{91438} \boldsymbol{\times}\mathbf{C} \boldsymbol{\times}\mathbf{D} \mathbf{-}\mathbf{0}\mathbf{.}\mathbf{37956} \boldsymbol{\times}\mathbf{A}^{\mathbf{2}}\mathbf{+}\mathbf{0}\mathbf{.}\mathbf{435439} \boldsymbol{\times}\mathbf{B}^{\mathbf{2}}\mathbf{-} \mathbf{2}\mathbf{.}\mathbf{31456} \boldsymbol{\times}\mathbf{C}^{\mathbf{2}}\mathbf{-} \mathbf{5}\mathbf{.}\mathbf{76956} \boldsymbol{\times}\mathbf{D}^{\mathbf{2}}$

Where, Y = RSY, A, B, C, D are Molar ratio, time, temperature, catalyst amount, respectively.

Significance of the models was investigated by applying “F-test” (Fisher’s test) using analysis of variance (ANOVA) to estimate the significance of the model, and based on obtained F-values, the highest order polynomial model with significant terms was selected (Table 1).

| **Source** | **Sum of squares** | **df** | **Mean square** | **F-value** | **p-value > F** |  |
| --- | --- | --- | --- | --- | --- | --- |
| Model | 5563.123 | 14 | 397.3659 | 49.56877 | < 0.0001 | Significant |
| A-Molar Ratio | 4984.013 | 1 | 4984.013 | 621.7226 | < 0.0001 |  |
| B - Time | 14.77867 | 1 | 14.77867 | 1.843542 | 0.1946 |  |
| C-Temperature | 58.50014 | 1 | 58.50014 | 7.297505 | 0.0164 |  |
| D-Amount | 1.817689 | 1 | 1.817689 | 0.226745 | 0.6408 |  |
| AB | 6.039306 | 1 | 6.039306 | 0.753363 | 0.3991 |  |
| AC | 5.164256 | 1 | 5.164256 | 0.644207 | 0.4347 |  |
| AD | 45.93451 | 1 | 45.93451 | 5.730026 | 0.0302 |  |
| BC | 18.59766 | 1 | 18.59766 | 2.319935 | 0.1485 |  |
| BD | 1.788906 | 1 | 1.788906 | 0.223154 | 0.6434 |  |
| CD | 13.37731 | 1 | 13.37731 | 1.66873 | 0.2160 |  |
| A^2 | 0.373264 | 1 | 0.373264 | 0.046562 | 0.8321 |  |
| B^2 | 0.491254 | 1 | 0.491254 | 0.061281 | 0.8078 |  |
| C^2 | 13.88 | 1 | 13.88 | 1.731439 | 0.2080 |  |
| D^2 | 86.24576 | 1 | 86.24576 | 10.75859 | 0.0051 |  |
| Residual | 120.2469 | 15 | 8.016457 |  |  |  |
| Lack of Fit | 120.2469 | 10 | 12.02469 |  |  | Not significant |
| Pure Error | 0 | 5 | 0 |  |  |  |
| Cor Total | 5683.37 | 29 |  |  |  |  |

**Table 1**: ANNOVA for FCCD results

Std. Dev. - 2.831335; Mean - 52.73033; C.V. (%) - 5.369461;R^2^ - 0.978842; Adj. R^2^ - 0.959095; Pred. R^2^ - 0.889956; df- Degrees of freedom; F-Fisher’s variance ratio; P-probability value; P<0.05- significant at 5 % level.

High F-value (49.56877) and lower P-value (probability, <0.0001) implies the model as significant for all the variables^1^. The “lack-of-fit” was found to be non-significant which accurately specified the model validity. Coefficient of determination (R^2^), which denotes the proportion of variance in the response values predictable from variation in interaction between process parameters was observed as 0.978842, further indicated the fitness and high statistical significance of the model with 97.88% variation in values of dependent variables (response) as shown in figure 5, wherein predicted and observed values of the response have been plotted for estimating the closeness of data, which found to be in the vicinity of the slope line.

**Figure 5**: Diagnostic plot for the experimental and predicted values of RSY for the parameters

The results obtained after conducting the complete set of 30 experiments in the form of observed values of response against coded values for acid-activated carbon is shown in Table 2. High values of adjusted R^2^ (0.959095) and predicted R^2^ (0.889956) for the quadratic model were further validated the significance of the model by showing a better correlation between the observed and predicted values.

| **Run No.** | **X1: Molar ratio** | **X2: Time (hour)** | **X3: Temperature (^o^C)** | **X4: Catalyst loading (wt. % w.r.t. glycerol)** | **Y:Response (% Conversion)** | |
| --- | --- | --- | --- | --- | --- | --- |
|  |  |  |  |  | **Experimental value** | **Predicted value** |
| 1. | 5 | 2 | 100 | 3 | 56.79 | 57.54728 |
| 2. | 8 | 3 | 150 | 1 | 65.05 | 65.04362 |
| 3. | 2 | 3 | 150 | 5 | 32.24 | 29.80917 |
| 4. | 2 | 1 | 50 | 5 | 37.27 | 37.81695 |
| 5. | 2 | 1 | 50 | 1 | 33.85 | 31.29515 |
| 6. | 8 | 3 | 50 | 1 | 70.04 | 70.11292 |
| 7. | 2 | 3 | 50 | 5 | 37.2 | 36.26348 |
| 8. | 5 | 2 | 100 | 3 | 56.79 | 57.54728 |
| 9. | 5 | 2 | 100 | 3 | 56.79 | 57.54728 |
| 10. | 2 | 1 | 150 | 1 | 33.48 | 32.81084 |
| 11. | 8 | 3 | 150 | 5 | 57.4 | 59.79292 |
| 12. | 5 | 2 | 150 | 3 | 55.76 | 53.42994 |
| 13. | 8 | 1 | 50 | 1 | 64.9 | 67.8714 |
| 14. | 5 | 3 | 100 | 3 | 58.68 | 57.07661 |
| 15. | 5 | 2 | 100 | 3 | 56.79 | 57.54728 |
| 16. | 5 | 2 | 100 | 1 | 53.35 | 51.45994 |
| 17. | 8 | 3 | 50 | 5 | 67.31 | 68.51973 |
| 18. | 8 | 1 | 150 | 1 | 66.34 | 67.11459 |
| 19. | 2 | 1 | 150 | 5 | 35.91 | 35.67515 |
| 20. | 5 | 2 | 100 | 5 | 51.72 | 52.0955 |
| 21. | 5 | 2 | 50 | 3 | 56.22 | 57.0355 |
| 22. | 2 | 3 | 50 | 1 | 31.41 | 31.07917 |
| 23. | 2 | 2 | 100 | 3 | 35.55 | 40.52772 |
| 24. | 5 | 2 | 100 | 3 | 56.79 | 57.54728 |
| 25. | 8 | 1 | 150 | 5 | 62.33 | 63.2014 |
| 26. | 5 | 1 | 100 | 3 | 58.8 | 58.88883 |
| 27. | 5 | 2 | 100 | 3 | 56.79 | 57.54728 |
| **28.** | **8** | **2** | **100** | **3** | **80.3** | **73.80772** |
| 29. | 2 | 3 | 150 | 1 | 26.65 | 28.28237 |
| 30. | 8 | 1 | 50 | 5 | 69.41 | 67.6157 |

**Table 2**: FCCD data from RSM for ketalisation reaction for acid activated carbon

Plotting a 3-D response surface between two independent variables is the best way to explicate their interaction effects on a dependent variable as shown in figure 6^2^.


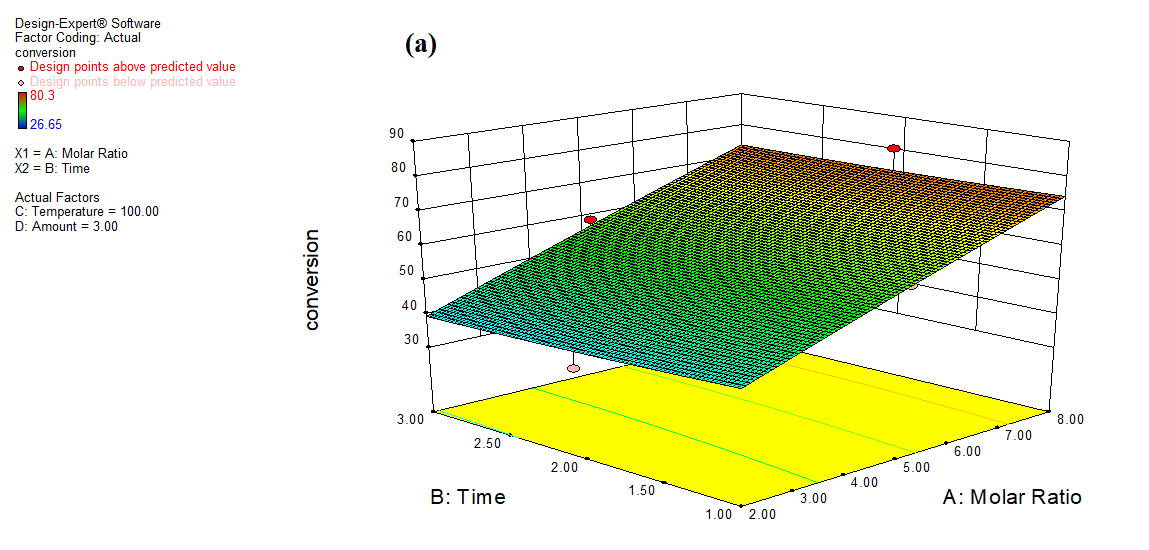


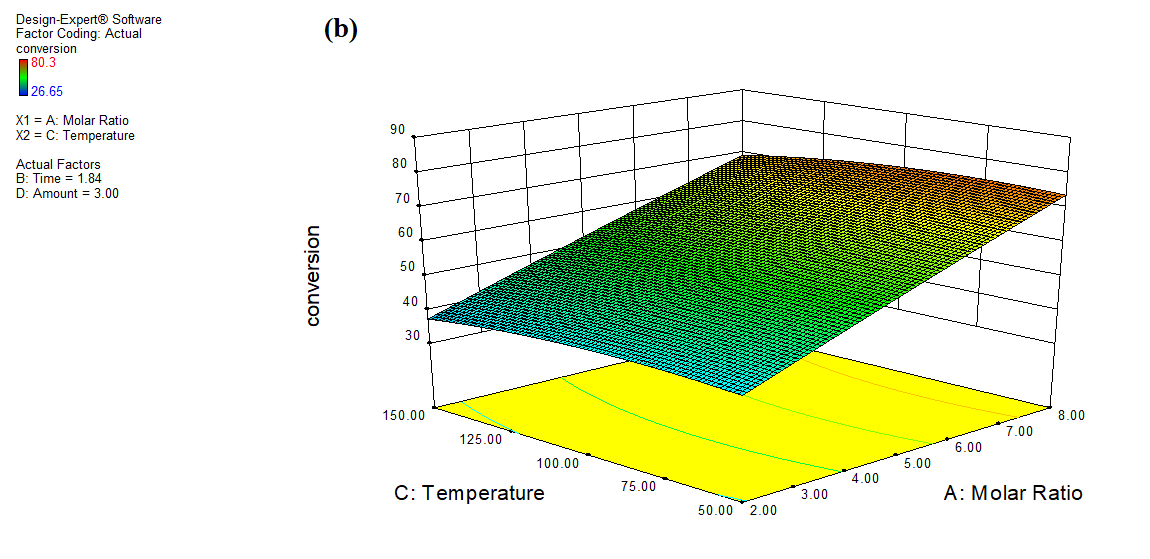


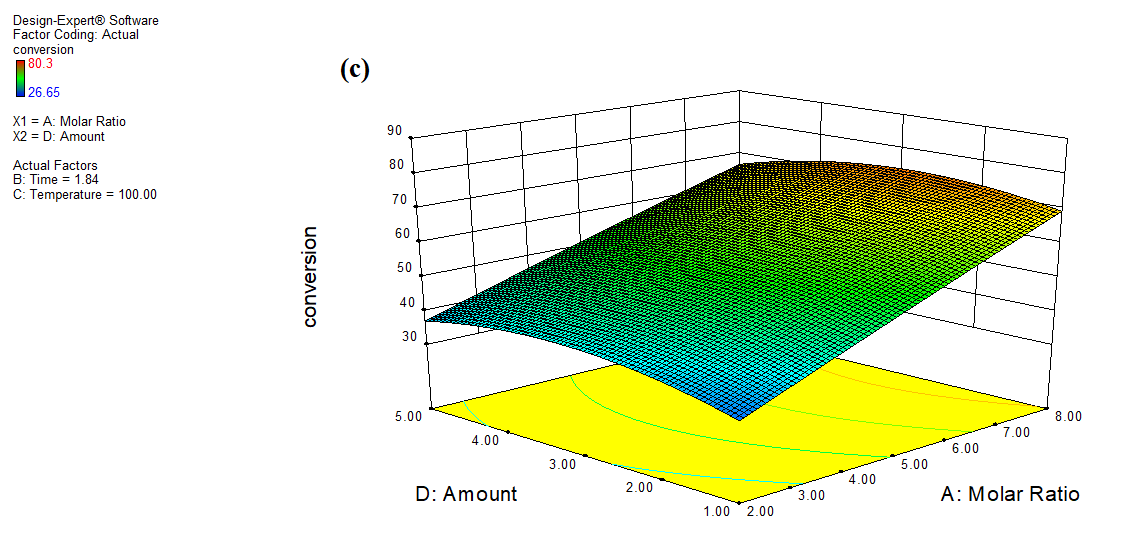


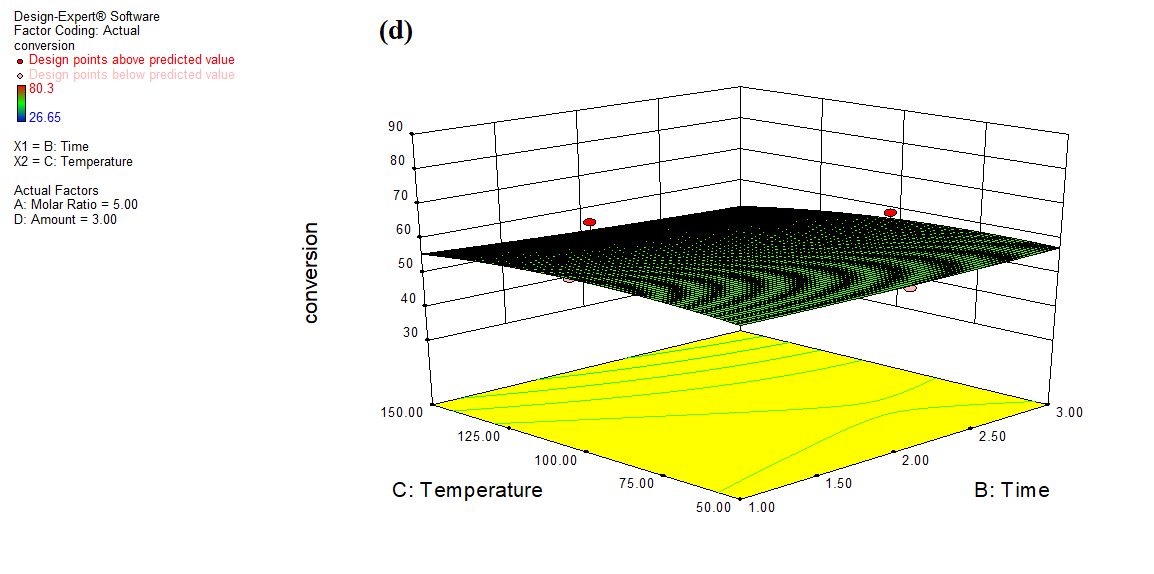


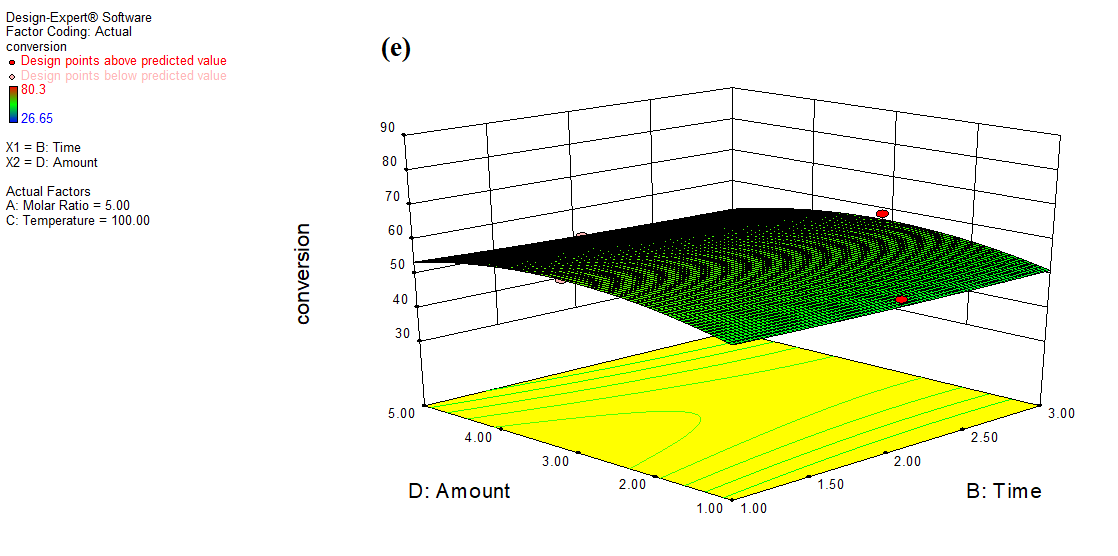


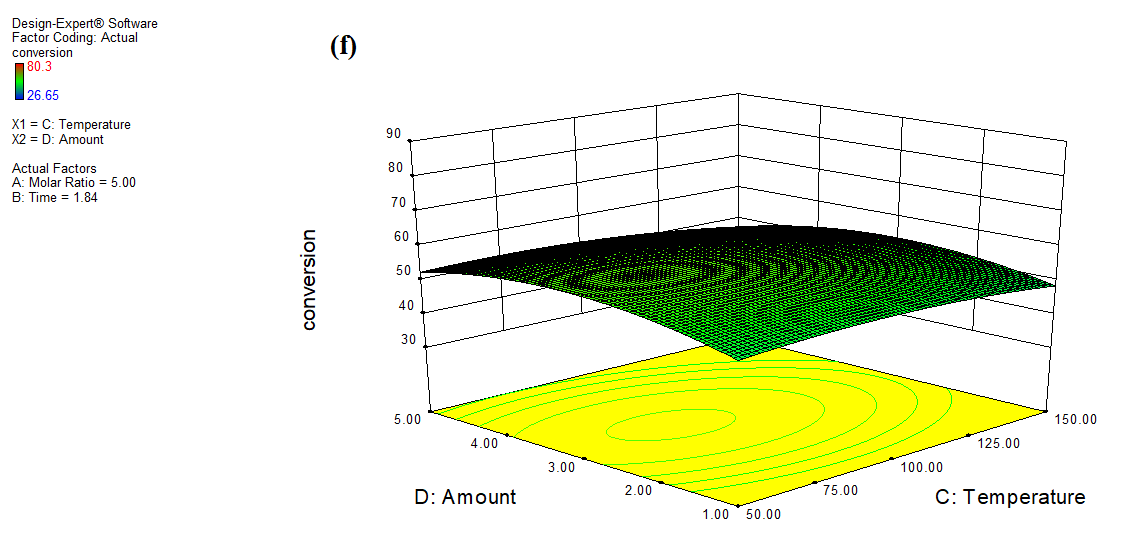


**Figure 6**: 3-D Plots for interaction between the process parameters for acid acivated carbon for glycerol conversion to produce solketal

**References**

[1] Arora R, Behera S, Sharma NK, Kumar S. A new search for thermotolerant yeasts, its characterization and optimization using response surface methodology for ethanol production. Front Microbiol. 2015 Sep 1;6:889. doi: 10.3389/fmicb.2015.00889.

[2] Y.H. Tan, M.O. Abdullah, C. Nolasco-hipolito, N.A. Zauzi, Application of RSM and Taguchi methods for optimizing the transesterification of waste cooking oil catalyzed by solid ostrich and chicken-eggshell derived CaO, Renew. Energy. (2017). https://doi.org/10.1016/j.renene.2017.07.024.
